# Utilizing maximal frequent itemsets and social network analysis for HIV data analysis

**DOI:** 10.1186/s13321-016-0184-9

**Published:** 2016-12-09

**Authors:** Yunuscan Koçak, Tansel Özyer, Reda Alhajj

**Affiliations:** 1 Department of Computer Engineering, TOBB University, Ankara, Turkey; 2 Department of Computer Science, University of Calgary, Calgary, Canada

## Abstract

Acquired immune deficiency syndrome is a deadly disease which is caused by human immunodeficiency virus (HIV). This virus attacks patients immune system and effects its ability to fight against diseases. Developing effective medicine requires understanding the life cycle and replication ability of the virus. HIV-1 protease enzyme is used to cleave an octamer peptide into peptides which are used to create proteins by the virus. In this paper, a novel feature extraction method is proposed for understanding important patterns in octamer’s cleavability. This feature extraction method is based on data mining techniques which are used to find important relations inside a dataset by comprehensively analyzing the given data. As demonstrated in this paper, using the extracted information in the classification process yields important results which may be taken into consideration when developing a new medicine. We have used 746 and 1625, Impens and schilling data instances from the 746-dataset. Besides, we have performed social network analysis as a complementary alternative method.

**Electronic supplementary material:**

The online version of this article (doi:10.1186/s13321-016-0184-9) contains supplementary material, which is available to authorized users.

## Background

Acquired immune deficiency syndrome (AIDS) is a deadly disease which is caused by human immunodeficiency virus (HIV). This virus destroys immune cells and yields patient’s body to become slowly defenseless against other diseases. According to Global Health Observatory (GHO) report, 78 million people are infected with HIV and it caused the death of 39 million pe ople. As of 2013, nearly 35 million people are living with HIV/AIDS and the mortality is 1.5 million [1]. There are various attempts to keep the virus under control. Unfortunately, effective cure has not been found yet despite the efforts to fully understand how the disease advances and its causes [2]. Inhibitors have been developed to keep it under control.

HIV-1 protease enzyme is used by the virus to cleave an amino acid octomer into peptides which are used to create essential proteins. These proteins are used by the virus to reproduce itself. Spread of the virus in the body is currently blocked with protease inhibitors. Herein, the main issue is to understand the link between HIV-1 protease and amino acid octomer for cleavage. Drugs become more of an issue during the therapy. Inhibitors mimic a peptide such that chemically modified peptide and scissile bond cannot be cleaved [15].

Available medicines work as HIV-1 protease inhibitors [3], i.e., the aim is to slow down reproduction of the virus. To design better inhibitors, it will be beneficial to find out amino acid sequences can be cleaved by HIV-1 protease [4]. This remains a difficult situation due to the uncertainty in patterns for cleavage sites of enzymes.

Amino acid residues are denoted by ${P_4}, {P_3}, {P_2}, {P_1},{P'_1},{P'_2},{P'_3},{P'_4}$ and their counterparts in protease are denoted by ${S_4},{S_3},{S_2},{S_1},{S'_1},{S'_2},{S'_3},{S'_4}$.

There are 20 possible amino acids which align to make an octamer. This leads to $20^8$ potential combinations of sequences. Data can be encoded in different ways. Although there are two alternative encoding schemes, namely OETMAP [5], and GP [6] encoding, it was noted in recent study by Rögnvaldsson et al. [13] that advanced feature encoding and selection schemes do not lead to better achievement in comparison to standard orthogonal encoding in samples without feature selection. A different Fresno-style approach was demonstrated by Liao et al. [29] who used Fresno semi-empirical scoring function to predict MHC molecule-peptide binding. Standard orthogonal encoding in representation has 160 binary positions (i.e., $20 \times 8$). While representing an octamer, out of 160 binary values, at each 20 bit length segment, one of them has value one to indicate an amino acid for the octamer. Hence, in total eight bits are set to one and 152 bits have value zero.

The problem of cleavage prediction resorts to binary classification from computational point of view. Recently, a consistency based feature selection mechanism associated with linear SVM has been proposed for the 746 dataset. Although there are several datasets as completely described in [13], some patterns for cleavage have been elicited particularly in the 746-dataset. In addition to SVM methods, neural networks [7] and markov models have been proposed in the literature. Another direction is introducing extra features by applying machine learning techniques. These techniques have been detailed in [13].

In this paper, we incorporate maximal frequent itemset mining to extract new features for cleavage prediction. These features have been added with different options to fully understand performance compared to results that use stand-alone standard encoding scheme. We alternatively utilize mining results for selected features which were previously named for the 746-dataset. Thus, we facilitate the use of social network analysis in feature selection. A social network graph is constructed based on results of the mining process. This forms a graph based on relationships among items (maximal frequent items). Actually, the power of social network analysis has been increasingly realized and the technique has gained huge interest in the research community. It became very popular in multi-disciplinary domains. Social network analysis focuses on relationships among social entities. The proposed methodology has been tested and the results reported in this paper demonstrate its applicability and effectiveness.

The rest of this paper is organized as follows. “The necessary background” section covers the background necessary to understand the approach described in this paper. In particular, we provide a brief overview of network analysis, fundamental definitions of frequent pattern mining and maximal frequent itemset mining. The proposed methodology is presented in “The methodology” section. Experiments and the analysis are discussed in “Experiment results and discussion” section; further, patterns specific to the 746-dataset have been used by using social network analysis. “Comparison of algorithms without feature selection” section is conclusions.

## The necessary background

The methodology described in this paper integrates techniques from social network analysis and data mining which are briefly covered in this section. We use frequent pattern mining to construct a network between various molecules.

### Social network analysis

A social network reflects connections between a set of items inspired from the investigated domain and called actors. Connections are determined based on the type of relationship to be studied and this may lead either to directed or to undirected network. A network may be analyzed based on existing actors and connections to reveal certain discoveries which may be valuable for effective and informative decision making.

Network analysis metrics includes a variety of measures which investigate various aspects of a given network. These include: (1) Degree centrality which is computed differently for directed and undirected networks. For the former, each node has in-degree and out-degree which are, respectively, number of links directed to and out of the node. For the latter, each node has a uniform degree which is the number of links connected to the node. (2) Betweenness centrality which is the number of shortest paths passing through a given node. (3) Density is the ratio of the number of links existing in a graph to the number of links in a complete graph, i.e., maximum density is one. (4) Eigen-vector centrality which determines how popular a given node is.

### Frequent patterns

Given a set of items. say *I*, it is possible to have various not necessarily disjoint subsets of *I* such that items in each subset are associated based on their coexistence in a given number of transactions where each transaction is a non-empty subset of *I*. Studying all associations across all subsets could reveal valuable information that describe some implicit relationships between various items. Items associated in a reasonable number of the given subsets form a frequent itemset. For instance, given genes in a body may be differently expressed in a number of samples forming different sets of expressed genes, one set per sample. These sets of expressed genes do overlap and analyzing them would lead to subsets of genes co-expressed together in a large number of samples. It is possible to determine a number of association rules from each frequent itemset by splitting the set into two non-empty disjoint subsets of the given itemset such that one subset forms the antecedent of the rule and the other subset forms the consequent of the rule. For instance, given a set of samples where only genes expressed in each sample are specified. $S_1: g_1, g_2,g_3,g_5,g_6$, $S_2: g_1.g_3,g_4, g_5, g_7$, $S_3: g_2,g_3, g_6, g_8$, and $S_4: g_1,g_3,g_4,g_8,g_9$. From these four samples, it is possible to find some frequent itemsets of co-expressed genes by assuming a minimum threshold value of 2, i.e., a set of genes is frequent if its genes coexist in at least 2 samples. An example frequent itemset could be {$g_1,g_3,g_4$}, {$g_2,g_3$}, etc.

Association rule mining has been well-studied in the literature [10]. Frequent itemsets are prominent for capturing intrinsic structure of a dataset. Formally speaking, given $T={t_1,t_2,\ldots ,t_n}$ as a dataset of *n* transactions, where each transaction $t_i$ contains items, e.g., $t_i = \{I_{i1},I_{i2},\ldots ,I_{ik}\}$ and each item $I_{ij} \in I$ the set of all possible items. An itemset, *IS* which contains items from *I*, is said to be frequent if and only if it is subset from a number of transactions in *T* greater than or equal to a pre-determined minimum support threshold value (*minsup*). Finally, given a set of items *F* an association rule is formally defined as $X\rightarrow Y$ such that $X\bigcup Y=F$, $X\ne \phi$, $Y\ne \phi$ and $X\bigcap Y=\phi$. An itemset *F* is characterized by support which is defined as the percentage of transactions from which *F* is subset. Further an association rule $X\rightarrow Y$ has a confidence value which is determined the fraction or ratio of support of $X\bigcup Y$ by support of *X*. Minimum support (*minsup*) and minimum confidence (*minconf*) threshold value are used in the minimg process for generating association rules that can be derived from *F*. Formally, support formula of itemset *F* is:

$$ support(F)=\frac{{{\# }\,\,{\text{of}}\,{\text{transactions}}\,{\text{having}}\,F}}{{|T|}} $$

where |*T*| is the total number of transactions. Itemset *F* is said to be frequent if and only if:

${\text{Frequent(F)}} = F \subseteq I\,\wedge \,{\text{support}}\,(F) \ge \,minsup$. Further, an association rule $X \rightarrow Y$ is said to be of specific importance when its confidence score is greater than or equal to minimum confidence value. Confidence formula is:

$$ {\text{confidence}}(X \rightarrow Y)= \frac{{\text{support(F)}}}{{\text{support(X)}}} $$

Frequent itemsets can be alleviated to different forms such as closed frequent itemsets [12] and maximal frequent itemsets [11]. A frequent itemset is closed if none of its supersets has its support. Formally,

$$ ClosedItemset(F) = {\text{Frequent(F)}} \,\wedge \, \forall Z ((Z \supset IS) \wedge (support(F) \ne support(Z))) $$

An itemset *F* is maximal, if it is frequent and none of its supersets is frequent. This can be formalized as:

$$ MaximalFrequentItemset(F) = {\text{Frequent(F)}} \,\wedge \, \forall Z ((Z \supset F) \wedge (frequent(Z) = False)) $$

Closed frequent and maximal frequent itemsets are two concise classes of itemsets which could be used to produced some valuable knowledge in a more controlled and efficient way as described in this paper.

## The methodology

Our methodology is organized in four phases. The first phase transforms the original input data by using orthogonal encoding. The second phase utilizes the new representation to find frequent itemsets from the new data representation. The third phase includes selecting the required itemsets from the obtained frequent itemsets. Finally, the selected itemsets are considered as features for classifying instances. Also as a complementary analysis, important itemsets are found by applying social network analysis metrics on a network among existing itemsets.

### Data modification

The methodology starts by transforming the original data into orthogonal encoding. In order to find frequent itemsets based on sequences of amino acid octomers, each amino acid is also changed to represent its position on the octomer. For example, the first instance of Schilling Dataset [17], namely

$$ AAAAAPAK $$

has been transformed into

$$ P_4A, P_3A, P_2A, P_1A, P'_1A, P'_2P, P'_3A, P'_4K. $$

In orthogonal encoding, there are 8 features for each instance and each feature can have 20 different values, one for each possible amino acid.

### Finding frequent itemsets

After transforming the dataset into the new representation, frequent itemsets based on the sequential amino acid octomer can be found. The FP-Growth algorithm has been used to extract frequent itemsets which are above a certain support threshold [18, 19]. In this study, maximal frequent itemsets have been extracted. Maximal frequent items give us a summarization of the given dataset. It is a lossy compression in the sense that all subsets of maximal itemsets are also frequent, but the support value of each subset itemset is not known.

In our experiments, the methodology works as follows: frequent itemsets are extracted in three ways by considering: (1) Data having cleavage class value, (2) Data having non-cleavage class value (3) All the dataset regardless of class value. These three frequent itemsets have been used in our experiments with different combinations.

The reason for separating the datasets is to find different patterns for different underlying class value. There may be some patterns that are frequent and specific to cleaving data. On the other hand, some other frequent itemsets may specific to data, and hence they are not cleaving. The separation leads to identifying all patterns, which may be in low support for the entire dataset whereas may have high support for a specific class value (cleavage or non-cleavage) without loss of generality.

### Alternatives for feature selection

We have accumulated number of features in terms of attribute patterns, which cover maximal frequent itemsets that are sufficient after selecting very low minimum support threshold value. In our experiments, we have determined this value as 0.05 which can be considered enough to capture enough number of itemsets.

Dataset features can be potentially expanded further, i.e., resorting to rich set of features. Then, the most informative features should be selected. During the process, frequent itemsets are used as features and the intersection between instances and features represents number of same amino acid occurrence at same residue. This function is named as *similarity*.

For example, assume *A* is a frequent itemset which contains items $(P'_3D, P'_1Y, P'_4S, P_1Y, P_4S)$. Assume *B* is an instance which consists of $(P_4A, P_3A, P_2A, P_1A, P'_1Y, P'_2P, P'_3D, P'_4K)$ amino acid octomer. The similarity between *A* and *B* is 2 because only items $P'_3D$ and $P'_1Y$ are present in both. The similarity formula can be expressed as:

$$ similarity(A,B) = number\, of\, same\, amino\, acid\, occurrence\, at\,same\, residue\, $$

The new dataset is constructed by applying the *similarity* function for every instance-feature combination. For a dataset with *M* instances and *N* frequent itemsets, the expansion of the dataset can have the size $M *N$.

In the first approach, we used the well known principal component analysis (PCA) technique. Briefly, it maps correlated features into linearly uncorrelated features. In other words, it can be used for dimensionality reduction. The second approach applies filtering by using a position based method. Here, frequent itemsets which have items at positions $P_1$ and $P'_1$ are selected. It has been reported that $P_1$ and $P'_1$ positions in octamer are important as they are informative to locate where cleavage happens. In this approach, only frequent itemsets containing items relevant to the mentioned positions have been considered [14]. The third approach utilizes social network analysis (SNA) methods for filtering. It is a novel feature selection method, which creates a social network between possible features. Then, for each feature in the network, its centrality score is calculated using different centrality measures. Consequently, features selected after applying the particular approach are introduced as the new dataset.

### Fitting into machine learning algorithm

We have rephrased the data in orthogonal encoding as suggested in [13]. Alternatively, a group of feature selection methods are proposed. After the feature selection process, the new dataset can be used for fit into classification to decide on the occurrence of cleavage. We have employed support vector machine (SVM) with linear kernel [13] and feature selection algorithms such as principal component analysis (PCA), RFE (Recursive Feature Elimination), Univariate ANOVA f value. Feature selection algorithms used 100 features in reduction. CMAR (JCBA) [25]^1^ and CPAR [26].^2^ ROC-AUC results are not reported for CPAR.

### Methodology of social network analysis

Social Network Analysis (SNA) is used to understand characteristics of a given network represented as a graph. Vertices represent actors in the network and edges represent interactions between actors.

By looking at network structure, it is possible to identify vertices which are more important compared to others. In general, vertices in the center of the network are more representative. As mentioned in “The necessary background” section, a variety of centrality measures are defined to reflect different perspectives by calculating different centrality scores of a vertex. One of these centrality metrics is normalized betweenness. Given a graph *G*, normalized betweenness centrality of a vertex *v* in *G* is calculated as the number of shortest paths passing through vertex *v* divided by total number of shortest paths in graph *G*. Another relevant centrality measure is PageRank [28], which is calculated as follows. After the initialization phrase, each vertex votes for other vertices regarding their importance and important vertices based on votes have higher impact for PageRank.

SNA measures have been used to find out which feature sets are more important for our problem. First, a social network of frequent itemsets is constructed. A matrix *M* was defined where each row represents an instance and each column represents a feature. The intersection between a row and a column is filled based on the *similarity* function defined in “Alternatives for feature selection” section.

$$ M_{ij} = similarity(M_i, M_j) $$

Given a two dimensional matrix which reflects a relationship between two sets of items (which are actors), folding is the process of multiplying a two dimensional matrix by its transpose to obtain a new matrix where rows and columns represent the same set of actors.Folding is applied on *M* to find similarity between frequent itemsets. Frequent itemsets form rows and columns of matrix *F* produced from the folding process.

$$ F=M^T \cdot M $$

After folding, a graph is constructed using adjacency matrix *F*, where each column is a vertex and if the entry at the intersection between a row and a column is greater than zero then an edge is constructed between the corresponding vertices. For this graph, PageRank and betweenness centrality measures are computed and the top 50 frequent itemsets are chosen.

## Experiment results and discussion

Four datasets have been utilized in the testing, namely 746Data [15], 1625Data [20], impensData [22–24] and schillingData [21]. Three of these datasets have been rectified (746Data, 1625Data, and schillingData) [13]. The four datasets are available at the UCI Machine learning repository,^3^ Details about these four datasets may be found in [13].

We have performed tenfold stratified cross validation technique for the classification in order to obviate with the overfitting problem. During the tenfold cross validation, for each test case, frequent itemsets have been found using all training folds, some frequent itemsets are selected and new dataset is created by applying similarity function over training and test instances (rows) and frequent itemsets (columns). The classifier model has been built using training folds and testing has been conducted using the remaining fold.

Our system has been implemented in python and using scikit-learn packages.^4^ SVC classifier has been used with linear kernel with penalty value as 1.0 and tolerance value for stopping criteria as 1e−4. Additionally, Pyfim^5^ has been used for extracting frequent itemsets. The cross validation results of the original dataset which were transformed into orthogonal encoding have been taken as baseline for comparison purposes. For the rest of the article, suggested methods are listed in Table 1.

**Table 1:** Abbreviation and explanation

Abbreviation	Explanation
OE	Orthogonally encoded features
FI-BOTH	Frequent itemsets are extracted from both cleaved and non-cleaved instances as features
FI-YES	Frequent itemsets are extracted only from cleaved instances as features
FI-NO	Frequent itemsets are extracted only from non-cleaved instances as features
SUP-m	Frequent itemset minimum support threshold m as percentage is used; if it is not present then 3% is used
PCA-100	Principal component analysis is used for feature selection and 100 features are selected
FI-CENTER	Frequent itemsets which have items in $P_1$ or $P^{\prime }_1$ position are selected as features
uni	ANOVA F value’s used for feature selection
RFE	Recursive feature elimination is used for feature selection
SNA-100	Social network analysis is used for feature selection and 100 features are selected

Table 1 lists abbreviations of steps and corresponding explanations. These abbreviations are used to explain which combination of the techniques mentioned in the methodology is used for experimentation. For example, OE + FI-BOTH + FI-CENTER + SUP-3 + PCA-100 stands for Orthagonal encoding and frequent itemsets which are extracted from both cleaved and non-cleaved instances with minimum support threshold 3% and only those having $P_1$ or $P'_1$ position as their items are used as features. Among all possible features principal component analysis is used to reduce dimensionality into 100 features.

To compare the performance of different combination of techniques, accuracy, precision, recall and F1-scores are calculated for all frequent itemset based experiments. F1-scores of experiments are compared and the one that has highest value is chosen as the best. This section is displayed in italic font.

**Table 2:** 746 Dataset—without feature selection

Methodology	Accuracy	Precision	Recall	F1	ROC-AUC
OE	0.869	0.871	0.910	0.883	0.956
FI-BOTH	0.869	0.904	0.860	0.869	0.956
OE + FI-BOTH	0.863	0.888	0.870	0.869	0.955
*FI-YES*	*0.887*	*0.905*	*0.897*	*0.896*	*0.962*
OE + FI-YES	0.871	0.891	0.882	0.878	0.958
FI-NO	0.873	0.904	0.877	0.880	0.949
OE + FI-NO	0.883	0.893	0.905	0.893	0.953
CMAR	0.789	0.812	0.79	0.783	0.777
CPAR	0.662	0.712	0.854	0.777	NA

Significant values are typed in italic

**Table 3:** 746 Dataset—with feature selection

Methodology	Accuracy	Precision	Recall	F1	ROC-AUC
OE + RFE-100	0.875	0.882	0.905	0.885	0.961
OE + UNI-100	0.861	0.865	0.902	0.876	0.95
OE + PCA-100	0.865	0.883	0.880	0.872	0.949
*OE + FI-BOTH + PCA-100*	*0.876*	*0.912*	*0.862*	*0.877*	*0.962*
OE + FI-YES + PCA-100	0.874	0.899	0.872	0.877	0.961
OE + FI-NO + PCA-100	0.873	0.904	0.865	0.875	0.957
OE + FI-BOTH + RFE	0.872	0.893	0.887	0.879	0.958
OE + FI-BOTH +CENTER RFE	0.875	0.888	0.892	0.883	0.957
OE + FI-YES + RFE	0.868	0.89	0.877	0.874	0.956
OE + FI-YES + CENTER RFE	0.865	0.887	0.877	0.872	0.956
OE + FI-NO + RFE	0.866	0.88	0.89	0.875	0.953
OE + FI-NO + CENTER RFE	0.887	0.893	0.912	0.897	0.960
OE + FI-BOTH + uni	0.859	0.888	0.863	0.866	0.935
OE + FI-BOTH + CENTER uni	0.842	0.867	0.862	0.855	0.934
OE + FI-YES + uni	0.840	0.868	0.850	0.851	0.927
OE + FI-YES + CENTER uni	0.864	0.893	0.870	0.871	0.938
OE + FI-NO + uni	0.866	0.887	0.885	0.878	0.950
OE + FI-NO + CENTER uni	0.860	0.872	0.885	0.869	0.951

Significant values are typed in italic

### 746 dataset

Results of the experiments on 746 Dataset are reported in Tables 2 and 3 without and with features selection, respectively. First of all, orthogonal encoding and orthogonal encoding with PCA reduction to 100 features are measured as base case. For Table 2, frequent itemsets are extracted for three different situations and their performance is measured. Then, orthogonal encoding features are added into frequent itemset features and the performance of this dataset is measured.

The first thing we noticed is that PCA selection reduces accuracy when only orthogonal encoding is used. Compared to orthogonal encoding, selecting frequent itemsets on cleaved instances (FI-YES) yield better results in terms of accuracy, f1 and ROC-AUC scores. FI-BOTH performed similar compared to OE. Among these three, FI-NO has the worst accuracy.

Combining orthogonal encoding features and frequent itemset features reported some interesting results. For FI-YES, this combination yields worse f1 score compared to using only FI-YES. For FI-BOTH, combining it with OE has no effect on f1 score. This can be explained as frequent itemsets derived from FI-YES and FI-BOTH can represent the dataset with similar degree compared to OE. For this reason, adding OE and FI-YES or FI-BOTH gives us worse or similar results by increasing dimensionality without adding much information. But, combining OE and FI-NO improves f1 score compared to using FI-NO only.

Table 3 shows the results of using feature selection. Among all features, 100 features are selected before testing the classifier using PCA, uni and RFE. It is important to note that all combinations of FI and OE with PCA yield better results compared to OE and PCA only. Lastly, best result of this experiment is achieved by combining OE, FI-NO and RFE-100. This score is also better than FI-BOTH and OE + FI-BOTH reported in Table 2.

**Table 4:** Impens dataset—without feature selection

Methodology	Accuracy	Precision	Recall	F1	ROC-AUC
OE	0.876	0.620	0.645	0.636	0.899
FI-BOTH	0.889	0.675	0.603	0.628	0.893
*OE + FI-BOTH*	*0.885*	*0.654*	*0.696*	*0.659*	*0.903*
FI-YES	0.866	0.609	0.656	0.609	0.890
OE + FI-YES	0.871	0.622	0.649	0.615	0.906
FI-NO	0.882	0.634	0.569	0.590	0.880
OE + FI-NO	0.879	0.625	0.67	0.634	0.900
CMAR	0.842	0.71	0.843	0.771	0.5
CPAR	0.552	0.277	0.83	0.416	NA

Significant values are typed in italic

**Table 5:** Impens dataset—with feature selection

Methodology	Accuracy	Precision	Recall	F1	Roc-Auc
OE + RFE 100	0.89	0.676	0.662	0.652	0.901
OE + UNI 100	0.881	0.662	0.616	0.625	0.901
OE + PCA-100	0.871	0.620	0.623	0.662	0.889
OE + FI-BOTH + PCA-100	0.891	0.672	0.596	0.627	0.893
OE + FI-BOTH + FI-CENTER + PCA-100	0.893	0.706	0.576	0.621	0.897
OE + FI-YES + PCA-100	0.881	0.650	0.596	0.611	0.886
OE + FI-YES + FI-CENTER + PCA-100	0.888	0.669	0.602	0.622	0.890
OE + FI-NO + PCA-100	0.879	0.640	0.570	0.593	0.880
OE + FI-NO + FI-CENTER + PCA-100	0.895	0.685	0.630	0.650	0.901
OE + FI-BOTH + RFE	0.886	0.663	0.61	0.625	0.890
*OE + FI-BOTH + FI-CENTER + RFE*	*0.896*	*0.678*	*0.675*	*0.666*	*0.911*
OE + FI-YES + RFE	0.880	0.661	0.662	0.641	0.910
OE + FI-YES + FI-CENTER + RFE	0.891	0.670	0.669	0.655	0.910
OE + FI-NO + RFE	0.879	0.634	0.649	0.631	0.892
OE + FI-NO + FI-CENTER + RFE	0.889	0.675	0.622	0.635	0.902
OE + FI-BOTH + uni	0.889	0.712	0.590	0.639	0.896
OE + FI-BOTH + FI-CENTER + uni	0.879	0.666	0.589	0.612	0.891
OE + FI-YES + uni	0.862	0.656	0.603	0.603	0.871
OE + FI-YES + FI-CENTER + uni	0.886	0.679	0.610	0.637	0.899
OE + FI-NO + uni	0.889	0.698	0.610	0.645	0.893
OE + FI-NO + FI-CENTER + uni	0.882	0.668	0.596	0.619	0.888

Significant values are typed in italic

### Impens dataset

Results for this dataset are reported in Tables 4 and 5, without and with feature selection. Compared to the base case, FI-BOTH and FI-NO have better accuracy but worse f1 score in Table 4. FI-YES has worst f1 score among all frequent itemset methods and CPAR has the worst f1 score of all experiments. In this dataset, using OE with frequent itemset methods improves f1 scores. In our experiments, using OE with FI-BOTH yields the best results among FI based methods and CMAR has the best results among all for Impens dataset without feature selection.

In feature selection case, experiments with frequent itemsets have higher accuracy compared to without feature selection counterpart. Also it is interesting to see that using FI-CENTER and selecting itemsets which have a $P_1$ or $P'_1$ position item, always increase accuracy and ROC-AUC score. Also F1 score increased for FI-YES and FI-NO. Biggest improvement happens with FI-NO and the best result among all feature selection experiments is using OE with FI-BOTH and filtering by FI-CENTER selected by RFE. This case also has better performance than the OE base case.

**Table 6:** 1625 Dataset—without feature selection

Methodology	Accuracy	Precision	Recall	F1	ROC-AUC
OE	0.929	0.885	0.820	0.839	0.980
FI-BOTH	0.926	0.876	0.823	0.836	0.977
FI-BOTH + SUP-1	0.925	0.877	0.817	0.836	0.980
OE + FI-BOTH	0.926	0.872	0.820	0.836	0.979
FI-YES	0.915	0.866	0.777	0.805	0.975
FI-YES + SUP-1	0.924	0.874	0.820	0.834	0.979
OE + FI-YES	0.923	0.874	0.807	0.828	0.980
FI-NO	0.922	0.860	0.820	0.825	0.976
*FI-NO + SUP-1*	*0.930*	*0.885*	*0.828*	*0.844*	*0.977*
OE + FI-NO	0.928	0.875	0.828	0.841	0.979
CMAR	80.9846	0.792	0.81	0.791	0.661
CPAR	0.813	0.674	0.941	0.785	NA

**Table 7:** 1625 Dataset—with feature selection

Methodology	Accuracy	Precision	Recall	F1	ROC-AUC
OE + RFE - 100	0.926	0.873	0.826	0.837	0.977
OE + UNI - 100	0.937	0.9	0.839	0.859	0.981
OE + PCA-100	0.927	0.866	0.841	0.843	0.978
OE + FI-BOTH + FI-CENTER + PCA-100	0.927	0.861	0.836	0.839	0.978
OE + FI-BOTH + FI-CENTER + SUP-1 + PCA-100	0.926	0.865	0.833	0.838	0.977
OE + FI-YES + FI-CENTER + PCA-100	0.920	0.859	0.812	0.823	0.977
OE + FI-YES + FI-CENTER + SUP-1 + PCA-100	0.923	0.869	0.823	0.833	0.977
OE + FI-NO + FI-CENTER + PCA-100	0.927	0.866	0.839	0.841	0.980
*OE + FI-NO + FI-CENTER + SUP-1 + PCA-100*	*0.934*	*0.887*	*0.839*	*0.852*	*0.979*
OE + FI-BOTH + RFE	0.923	0.860	0.815	0.827	0.973
OE + FI-BOTH + CENTER + RFE	0.927	0.881	0.817	0.837	0.977
OE + FI-BOTH + CENTER + RFE - sup1	0.929	0.885	0.82	0.841	0.978
OE + FI-YES + RFE	0.925	0.884	0.802	0.830	0.979
OE + FI-YES + CENTER + RFE	0.924	0.879	0.807	0.828	0.98
OE + FI-YES + CENTER + RFE - sup1	0.92	0.863	0.804	0.822	0.98
OE + FI-NO + RFE	0.925	0.853	0.839	0.837	0.973
OE + FI-NO + CENTER + RFE	0.919	0.851	0.815	0.823	0.976
OE + FI-NO + CENTER + RFE - sup1	0.924	0.855	0.833	0.835	0.975
OE + FI-BOTH + uni	0.894	0.854	0.653	0.712	0.951
OE + FI-BOTH + CENTER + uni	0.907	0.867	0.723	0.765	0.967
OE + FI-BOTH + CENTER + uni-sup1	0.897	0.849	0.674	0.719	0.953
OE + FI-YES + uni	0.898	0.846	0.704	0.734	0.958
OE + FI-YES + CENTER + uni	0.897	0.844	0.704	0.737	0.955
OE + FI-YES + CENTER + uni - sup1	0.911	0.866	0.75	0.778	0.972
OE + FI-NO + uni	0.907	0.866	0.737	0.776	0.966
OE + FI-NO + CENTER + uni	0.93	0.879	0.825	0.843	0.98
OE + FI-NO + CENTER + uni - sup1	0.895	0.827	0.701	0.732	0.961
OE + FI-BOTH + PCA-100	0.918	0.849	0.812	0.817	0.975
OE + FI-YES + PCA-100	0.920	0.859	0.812	0.823	0.977
OE + FI-NO + PCA-100	0.915	0.855	0.801	0.81	0.972

Significant values are typed in italic

### 1625 dataset

Results for this dataset are given in Tables 6 and 7. For without feature selection case, using only frequent itemsets based methods performed worse compared to base case in terms of f1 score and accuracy. Combining OE and frequent itemsets based methods improved performance for FI-YES and FI-NO. We also tried changing minimum support threshold to observe the change in accuracy and f1 score. Minimum support threshold for choosing maximal frequent itemsets have been changed from 3 to 1%. For FI-YES and FI-NO this change improved performance significantly and FI-NO with SUP-1 is our best result among all.

For feature selection case, FI-CENTER is applied to the combination of OE and frequent itemsets based methods. With only this addition, it couldn’t perform better than base case. After realizing the positive outcome of changing minimum support threshold, it was decreased to 1 percent. This change increased performance for FI-YES and FI-NO and most notable change happened for FI-NO. By applying this change, better results than base case were reported.

**Table 8:** Schilling dataset—without feature selection

Methodology	Accuracy	Precision	Recall	F1	ROC-AUC
OE	0.907	0.706	0.683	0.661	0.941
*FI-BOTH*	*0.922*	*0.774*	*0.623*	*0.668*	*0.949*
FI-YES	0.904	0.714	0.653	0.645	0.938
FI-NO	0.918	0.754	0.607	0.650	0.949
CMAR	0.867	0.752	0.867	0.806	0.5
CPAR	0.488	0.189	0.857	0.310	NA

Significant values are typed in italic

**Table 9:** Schilling dataset—with feature selection

Methodology	Accuracy	Precision	Recall	F1	ROC-AUC
OE + RFE 100	0.911	0.71	0.702	0.675	0.943
OE + UNI 100	0.911	0.697	0.69	0.673	0.939
OE + PCA-100	0.886	0.581	0.631	0.614	0.920
OE + FI-BOTH + FI-CENTER + PCA-100	0.926	0.765	0.663	0.699	0.941
*OE + FI-YES + FI-CENTER + PCA-100*	*0.931*	*0.797*	*0.665*	*0.715*	*0.951*
OE + FI-NO + FI-CENTER + PCA-100	0.927	0.777	0.667	0.701	0.945
OE + FI-BOTH + RFE	0.911	0.718	0.667	0.66	0.941
OE + FI-BOTH + FI-CENTER + RFE	0.912	0.719	0.692	0.672	0.948
OE + FI-YES + RFE	0.909	0.703	0.695	0.674	0.944
OE + FI-YES + FI-CENTER + RFE	0.911	0.695	0.697	0.679	0.942
OE + FI-NO + RFE	0.913	0.72	0.676	0.668	0.939
OE + FI-NO + FI-CENTER + RFE	0.911	0.712	0.681	0.667	0.945
OE + FI-BOTH + uni	0.9	0.655	0.619	0.612	0.924
OE + FI-BOTH + FI-CENTER + uni	0.908	0.679	0.692	0.668	0.936
OE + FI-YES + uni	0.876	0.605	0.579	0.56	0.896
OE + FI-YES + FI-CENTER + uni	0.892	0.639	0.618	0.605	0.913
OE + FI-NO + uni	0.93	0.879	0.825	0.843	0.98
OE + FI-NO + FI-CENTER + uni	0.909	0.686	0.697	0.672	0.937
OE + FI-BOTH	0.908	0.706	0.683	0.66	0.94
OE + FI-NO	0.907	0.707	0.676	0.655	0.94
OE + FI-YES	0.9	0.712	0.706	0.677	0.941
OE + FI-BOTH + PCA-100	0.926	0.765	0.663	0.699	0.941
OE + FI-YES + PCA-100	0.931	0.797	0.665	0.715	0.951
OE + FI-NO + PCA-100	0.911	0.728	0.547	0.6	0.917

Significant values are typed in italic

### Schilling dataset

Results for this dataset are reported in Tables 8 and 9. For this dataset, FI-NO performed better than FI-YES, but the best performing method is FI-BOTH among FI methods. Also FI-BOTH performed better than base case and overall CMAR has the highest f1 score.

For feature selection case, all frequent itemsets combined methods performed better when compared to FI only counterpart. Among them, combining OE with FI-YES and filtering by FI-CENTER performed the best as demonstrated by the reported results.

### Characteristics of patterns sfter RFE ranking

We have performed experiments for ranking features with RFE. We have applied three different approaches. The first approach considers adopting both cleavage and non-cleavage training data for frequent itemset generation, the second approach considers only cleaving training data for frequent itemset generation, and the last approach considers only non-cleaving training data. The results are summarized in Tables 10, 11, and 12

**Table 10:** Top ten patterns obtained with OE including frequent itemsets after RFE (FI-BOTH RFE) with (cleavage, non-cleavage) distribution

746	1625	Impens	Schilling
xx, (23,1)	xx, (40,4)	xxxxxxxR, (2,130)	xxxxxxxK, (5,382)
ARxLxEAx, (20,2)	PxxxLAMT, (42,0)	xxx, (0,41)	xxx, (0,218)
PAxxLAMT, (20,2)	Exx, (48,4)	xxx, (19,19)	xxx, (2,210)
S, (22,0)	xx, (39,2)	xxx, (3,94)	xxxxxxPx, (2,173)
xxVxFxxx, (23,1)	xxx, (52,28)	xxx, (18,15)	xxx, (2,197)
AxVxxxAM (16,6)	xAx, (39,12)	xxx, (15,15)	xxx, (4,139)
ARxLAExx, (16,3)	xxx, (24,24)	xxx, (0,60)	xxx, (1,182)
xxx, (0,20)	xxxxxxPx, (0,55)	xxx, (5,95)	xxx, (1,169)
TKxxxVQP, (17,3)	xxx, (0,95)	xxx, (1,60)	xxx, (2,83)
AxVLxxxM, (15,6)	xxx, (0,81)	xxx, (0,24)	xxx, (133,213)

We have composed top ten features for the datasets after RFE ranking of OE FI-BOTH in Table 10. The results indicate that for 746 dataset, nine out of ten are mostly observed as majority for cleavage instances. For 1625 dataset, six of them are cleavage and three of them are non-cleavage instances; one pattern is equally distributed between both. For impens and schilling datasets, 1-item frequent items are ranked in the first ten where percentage of cleavage dataset is higher for one of them; equal for cleavage and non-cleavage. Remaining eight patterns are mostly observed for non-cleavage datasets. For schilling dataset, all are mostly seen in non-cleavage data. Impens and schilling patterns are located close to center. It would be reasonable to pay attention to non-cleavage patterns mostly for development of inhibitors.

**Table 11:** Top ten patterns obtained with OE including frequent itemsets after RFE (FI-YES RFE) with (cleavage, non-cleavage) distribution

746	1625	Impens	Schilling
xxxFxExx (12,0)	PxVSLAMT (10,0)	xEx (4,20)	xx (15,2)
SQxYYxxx (11,0)	SQxYYxxx (11,0)	xExRxxxx (4,0)	xFx (14,5)
PxVxLAMT (27,0)	AxVLAEAx (13,0)	xx (4,0)	Exx (16,14)
xKxLVVQP (14,1)	TxxLVVQP (14,1)	xxIxYxxx (4,1)	xx (13,1)
x (14,0)	xx (11,1)	xxxxxEYx (4,0)	xx (13,21)
Exx (13,0)	PxxWLAMT (10,0)	xx (4,0)	Ixx (12,15)
xxNxPQxx (12,0)	SxTYYxDS (11,0)	xx (4,1)	xxxxIxLx (12,4)
SDTYYxxS (11,0)	xGx (10,0)	xx (6,3)	Pxx (12,8)
xQNYPIVQ (11,0)	SxxYYTDS (11,0)	xxxLxLxx (5,2)	xFExxxxx (13,3)
SxNxPxVQ (11,3)	SGxxxxxS (11,1)	xWxxxxxx (4,0)	xxx (36,37)

In Table 11, frequent itemsets obtained from cleaved training data have been used for feature ranking and top ten patterns have been presented for the datasets. Majority of patterns are attributed to cleaving data in the entire dataset. Itemsets that contain six or 7 items are ranked in the first ten patterns. They are not listed in FI-BOTH experiment. Itemsets containing more than one item occur in Impens and Schilling datasets. Nine of them occur more in cleaving instances and one in non-cleaving instances. It may be surprising that one pattern obtained in FI-YES may exist in non-cleaving instances but occurrence of same pattern in non-cleaving instances is possible. This is similar for Schilling dataset. Eight patterns exist mostly in cleaving instances and two exist in non-cleaving instances. Patterns represent instances having significant positions closer to center.

**Table 12:** Top ten patterns obtained with OE including frequent itemsets after RFE (FI-NO RFE) with (cleavage, non-cleavage) distribution

746	1625	Impens	Schilling
Axx (46,9)	xxx (0,97)	xxx (8,95)	xxx (133,213)
xxx (86,11)	xxx (0,95)	xxR (0,20)	xxx (1,168)
xxx (0,20)	xxx (5,81)	xx (1,21)	xxx (1,82)
xxx (66,10)	xxx (0,61)	xxx (0,45)	xxx (0,86)
xxx (1,15)	xxx (98,43)	xxx (0,23)	xxx (5,252)
Sxx (76, 9)	xxxxxxKx (2,95)	xxx (2,45)	xxxxxxxK (5,382)
SxxxxxNx (8,10)	xxx (1,69)	xxx (4,29)	xxxxxxxR (1,172)
xxxxxxxT (98,17)	xxx (0,76)	xxx (0,41)	xxx (2,197)
xxx (0,13)	xxx (3,83)	xxx (1,68)	xxx (1,169)
xxx (0,17)	xxx (0,57)	xxx (2,40)	xxx (0,73)

In Table 11, non-cleaved instances have been mined to extract frequent patterns from non-cleaving instances(FI-NO). Top ten patterns have been listed for the datasets. It is noticeable that patterns are mostly 1-item frequent itemsets. The reason is that we were unable to find characteristic patterns for non-cleavage since they are collected from dispersed space. For 746 dataset, 5 cleavage and 5 non-cleavage; for 1625 dataset, one cleavage and nine non-cleavage and for Impens and Schilling datasets; all patterns are mostly found in non-cleavage instances. Again, patterns closer to center are identified as significantly top ranking patterns.

### Complementary analysis by social network analysis

**Fig. 1:**
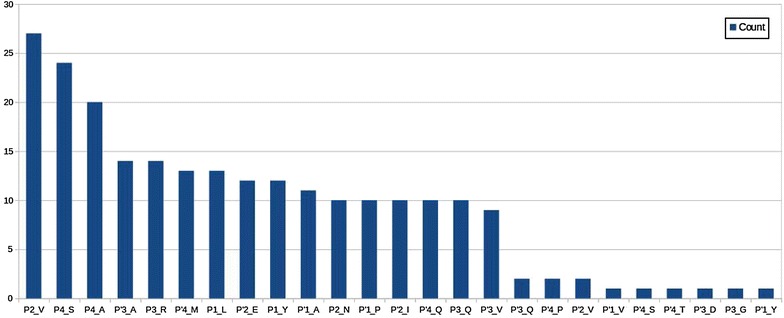
Histogram for 50 features selected by PageRank centrality

**Fig. 2:**
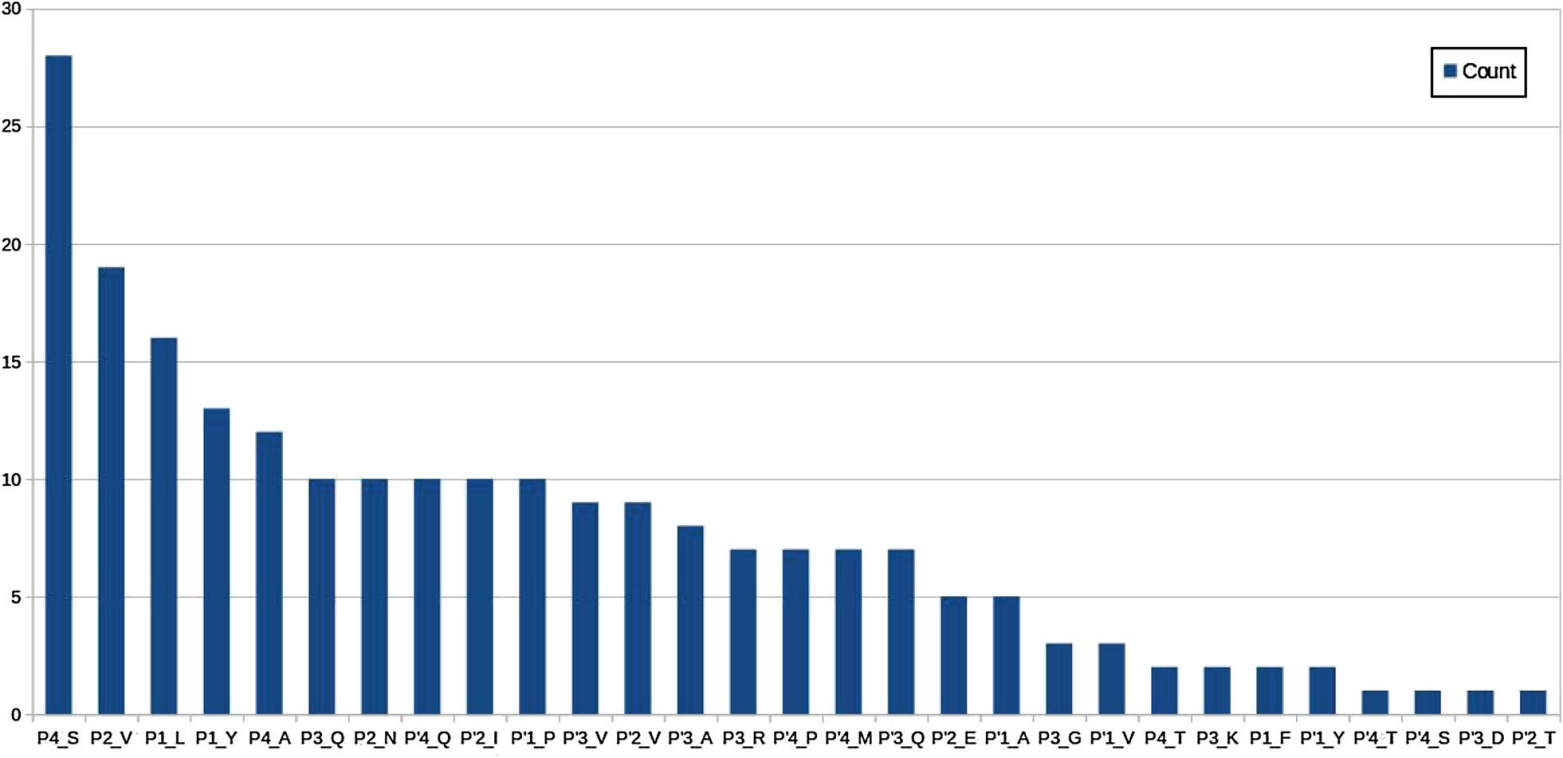
Histogram for 50 features selected by betweenness centrality

**Fig. 3:**
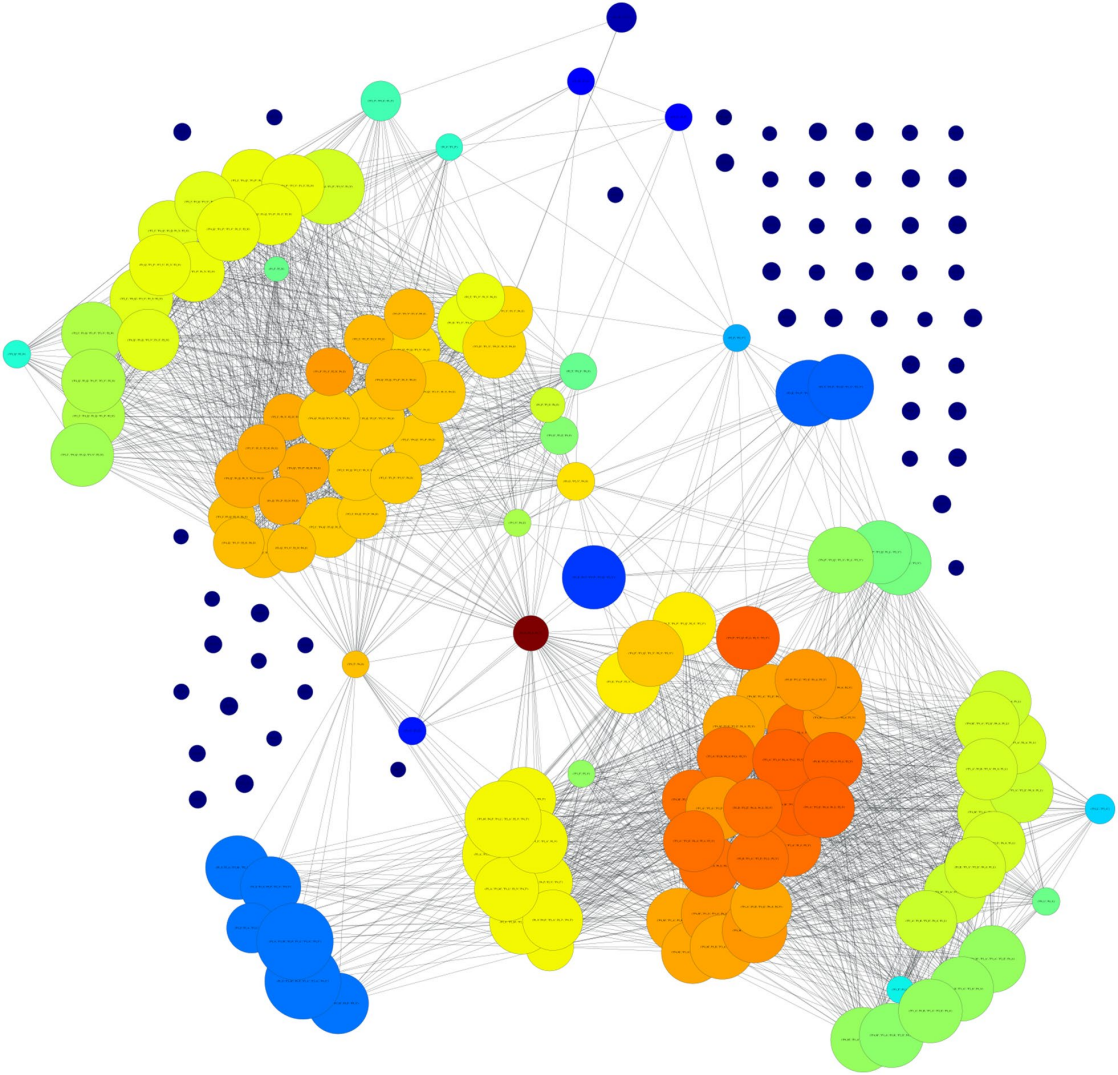
Graph for network colored by PageRank

For the network created based on frequent itemsets, PageRank and betweenness centrality measures are computed and the top 50 frequent itemsets are chosen. Histograms are created to understand the distribution of the selected features. Histograms of each item are shown in Figs. 1 and 2, for pagerank and betweenness, respectively. Figure 3 visualizes the network where color has been determined according to pagerank centrality values using jet colormap. Figue 4 displays the network where color has been determined according to betweenness centrality values using jet colormap. Finally, top five features are reported in Tables 13 and 14 for pagerank and betweenness, respectively.

**Fig. 4:**
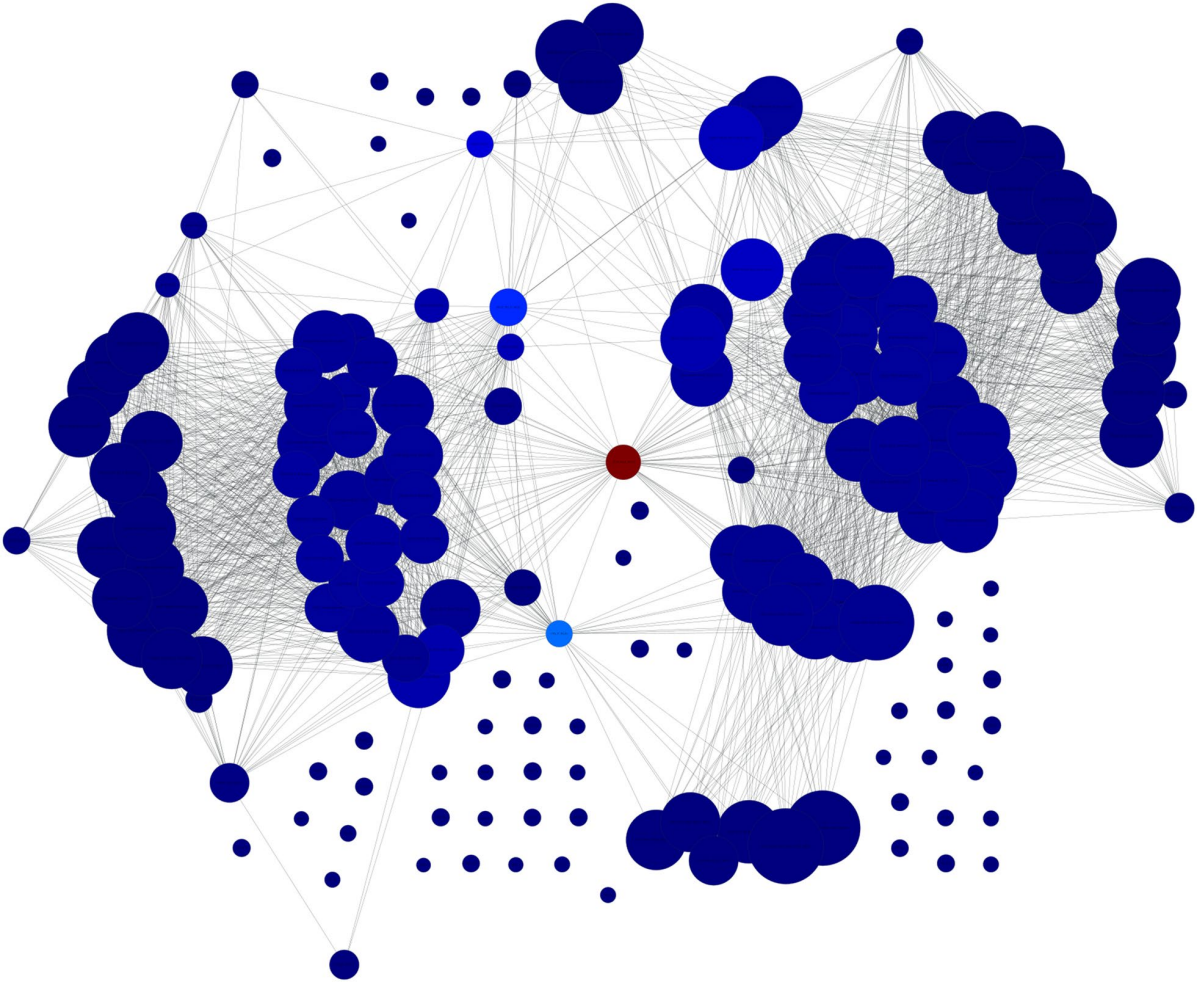
Graph for network colored by betweenness

**Table 13:** Top 5 vertices according to PageRank

Vertex	Centrality score
$(P_3G, P_4S, P_2V)$	0.0088053091045
$(P'_4P, P'_3Q, P_1L, P_2V, P'_2V)$	0.00729830131176
$(P'_4M, P'_3A, P_4A, P_1L, P_2V)$	0.00726810634265
$(P'_1A, P'_3A, P_4A, P_1L, P_2V)$	0.00726810634265
$(P_3R, P'_3A, P_4A, P_1L, P_2V)$	0.00726810634265

**Table 14:** Top 5 vertices according to betweenness

Vertex	Centrality score
$(P_3G, P_4S, P_2V)$	0.152425782917
$(P'_4T, P_4S)$	0.0356197541186
$(P_3G, P'_2V, P_4S)$	0.0254409174283
$(P_1F, P'_2V)$	0.0119543726703
$(P'_4P, P'_3Q, P_1L, P_2V, P'_2V)$	0.00917331679147

**Table 15:** Intersection between selected features

Methodology	SNA betweenness	SNA pagerank
C-FS-SVM	5	4
Column Ccnsistency	9	8
Column SVM	7	5
Consistency	9	8
Consistency-SVM	5	4
FS-MLP	5	4

**Table 16:** Intersected features between selected features

Methodology	SNA betweenness	SNA pagerank
C-FS-SVM	$(P_1Y, P_1L, P'_4T, P_1F, P_2V)$	$(P_1Y, P_1L, P'_4T, P_2V)$
Column consistency	$(P_2N, P'_3Q, P_1F, P_1Y, P_4S, P'_4P, P'_4T, P'_3D, P_1L)$	$(P_2N, P'_3Q, P_1Y, P_4S, P'_4P, P'_4T, P'_3D, P_1L)$
Column SVM	$(P_1Y, P_3K, P'_2E, P'_4T, P_1F, P_2V, P_1L)$	$(P_1Y, P_1L, P'_4T, P'_2E, P_2V)$
Consistency	$(P_3G, P'_3Q, P_1Y, P_4S, P'_2E, P_1F, P_2V, P_1L, P'_2V, )$	$(P_3G, P'_3Q, P_1Y, P'_2E, P_4S, P_2V, P_1L, P'_2V)$
Consistency-SVM	$(P_1Y, P_1L, P_1F, P'_2E, P_2V)$	$(P_1Y, P_1L, P'_2E, P_2V)$
FS-MLP	$(P_2N, P_1L, P_1F, P'_2E, P_1Y)$	$(P_2N, P_1L, P'_2E, P_1Y)$

The selected features are compared with the results reported in [14]. Authors of [14] have worked with 754 dataset; for this reason, we compared our findings from 746 dataset. Intersection of selected features and intersection amount are presented in Tables 15 and 16.

## Comparison of algorithms without feature selection

We have used Keel application [27] to estimate effectiveness of our algorithm.^6^ Average ranks have been obtained by applying Friedman procedure.

**Table 17:** Average ranking of the algorithms without feature selection

Algorithm	Ranking
OE	3.25
FI-BOTH	4.75
OE + FI-BOTH	4.5
FI-YES	6
OE + FI-YES	5
FI-NO	6.25
OE + FI-NO	3.5
CMAR	4.5
CPAR	7.25

Table 17 summarizes the f-score ranking of algorithms having no feature selection. Ranking has been computed with Friedman statistic with $(9-1)$ degrees of freedom and distribution of chi-square as 7.2. P value computed by Friedman test was 0.515.

Based on the results, recommended orthogonal encoding scheme with SVM classifier performs the best [13]. Overall analysis of algorithms indicate that OE with SVM performs the best with overall ranking result value 3.25. OE + FI-NO ranks second algorithm with overall ranking result value, which is 3.5. Next two algorithms OE + FI-BOTH and CMAR get the third place with value as 4.5. FI-BOTH value is 4.75. OE + FI-YES is 5, and FI-YES value is 6. CPAR has the worst ranking overall. According to the null hypothesis, all classifiers have no difference; this is rejected since they are not equal.

Later, We have performed $$N *N $$ post hoc comparison with Shaffer’s statistical test. Additional file 1: Table S1 gives comparison results between algorithms. In this table, *p* and adjusted Shaffer *p* value as the adjusted value are listed. Comparison results give p values which when higher favor the null hypothesis that claims that the compared two algorithms are not significantly different.

**Table 18:** Average rankings of the algorithms

Algorithm	Ranking
OE	9.125
OE + PCA-100	10.125
OE + FI-BOTH + PCA-100	10.75
OE + FI-BOTH + FI-CENTER + PCA-100	9.375
OE + FI-YES + PCA-100	11
OE + FI-YES + FI-CENTER + PCA-100	9
OE + FI-NO + PCA-100	18.125
OE + FI-NO + FI-CENTER + PCA-100	3.375
OE FI-BOTH RFE	12.25
OE FI-BOTH CENTER RFE	6
OE FI-YES RFE	10
OE FI-YES CENTER RFE	9
OE FI-NO RFE	11.375
OE FI-NO CENTER RFE	10
OE FI-BOTH uni	17.25
OE FI-BOTH CENTER uni	18
OE FI-YES uni	21.5
OE FI-YES CENTER uni	17
OE FI-NO uni	12.25
OE FI-NO CENTER uni	12.25
OE+RFE	6
OE+UNI	9.25

We repeated the statistical analysis for algorithms with feature selection including OE with SVM and the first one bundled with feature selection algorithms such as RFE and univariate Anova analysis. Table 18 summarizes the f-score ranking of algorithms having no feature selection. Ranking has been computed with Friedman statistic with $(22-1)$ degrees of freedom and distribution of chi-square as 39.139328. P-value computed by Friedman test was 0.009445911037720411. Based on the results, ranking of the listed algorithms are different and OE + FI-NO + FI-CENTER + PCA-100 outperforms OE and OE + RFE. Its ranking value is 3.375. The second ranked algorithms are OE FI-BOTH CENTER RFE and OE + RFE(6). OE has the value 9.125.

Later, we performed $$N *N $$ post hoc comparison with Shaffer’s statistical test. Additional file 1: Table S2 gives comparison results between algorithms. This table lists *p* and adjusted Shaffer *p* value as the adjusted value. Comparison results give p values which when higher favor the null hypothesis that claims that the compared two algorithms are not significantly different.

## Conclusions and future work

AIDS is a deadly disease caused by HIV. Cleaving proteins is an important event for HIV. Understanding patterns for this process will lead to improvements on drug design. The proposed approach views HIV data from a different perspective, where features are enriched with frequent itemsets, with support values with respect to their occurrences within the training data. Hence, features are reorganized at each section in cross validation. This is a novel approach in terms of feature extraction and dimensionality reduction. Our approach to tackle this problem was to extract frequent itemsets based on sequential amino acids in octomer. Three different sets of maximal frequent itemsets are extracted based on cleave property of an instance. These maximal frequent itemsets are used as features and the intersection of instance and feature are filled according to similarity function. After this process, a dataset is fit into the machine learning algorithm and results are reported.

Our results show that using frequent itemsets as features has positive impact on performance. For some cases, using only frequent itemsets as features can represent a dataset better than OE. For other cases, frequent itemsets features can be used as supplementary features which also improved performance compared to OE. In most cases, feature selection among the combination of OE and FI-based methods yields better performance and using less features compared to OE. Minimum support threshold is also an important parameter for FI-based methods, changing it can lead to increased performance.

Our complementary analysis benefits from itemsets to generate a network which will help in finding important features by using SNA metrics described in the literature. In general, they are used to understand dynamics of social networks. Particularly, in our work, it is used to understand the relationship between residues and amino acid groups. For top 50 features, histograms of items are presented. Top 5 features are reported and graph is visualized to see the influence between features. Also the chosen features are compared with another work and similarities between selected features are shown. All these results demonstrate effectiveness of the proposed methodology.

In-depth analysis for making biological explanation remains another future direction. In the future, fascicles of different domains on molecular biology will be studied.

## Additional file

**Additional file 1.** Non-parametric statistic methods for comparative analysis between methods.

## Footnotes

1
http://weka.sourceforge.net/doc.packages/classAssociationRules/weka/classifiers/rules/car/JCBA.html.

2
https://github.com/d2fn/cpar-classifier.

3
https://archive.ics.uci.edu/ml/datasets/HIV-1+protease+cleavage.

4
http://scikit-learn.org/stable/.

5
http://www.borgelt.net/pyfim.html.

6
http://www.keel.es/algorithms.php.
