# Supplementary material for: Utilizing maximal frequent itemsets and social network analysis for HIV data analysis
Source: J Cheminform. 2016 Dec 9;8:71. doi: 10.1186/s13321-016-0184-9 (PMC5395515; doi:10.1186/s13321-016-0184-9)
Supplement: Supplementary file 1 — Additional file 1. Non-parametric statistic methods for comparative analysis between methods. [file 13321_2016_184_MOESM1_ESM.pdf]

Supplementary Material: Non-Parametric Statistic Methods for Comparative Analysis Between Methods

1 Post hoc comparison with Shaffer's statistical test results

Post hoc comparison results of algorithms with and without feature selection is presented.

i	hypothesis	unadjusted p	p_{Shaf}
1	OE vs .CPAR	0.038867	1.399216
2	OE + FI-NO vs .CPAR	0.052808	1.47861
3	OE vs .FI-NO	0.121335	3.397387
4	OE vs .FI-YES	0.15558	4.35625
5	OE + FI-BOTH vs .CPAR	0.15558	4.35625
6	FI-NO vs .OE + FI-NO	0.15558	4.35625
7	CMAR vs .CPAR	0.15558	4.35625
8	FI-BOTH vs .CPAR	0.196706	5.507757
9	FI-YES vs .OE + FI-NO	0.196706	5.507757
10	OE + FI-YES vs .CPAR	0.245278	5.507757
11	OE vs .OE + FI-YES	0.366157	8.055444
12	OE + FI-BOTH vs .FI-NO	0.366157	8.055444
13	FI-NO vs .CMAR	0.366157	8.055444
14	OE vs .FI-BOTH	0.438578	9.648717
15	FI-BOTH vs .FI-NO	0.438578	9.648717
16	OE + FI-BOTH vs .FI-YES	0.438578	9.648717
17	FI-YES vs .CMAR	0.438578	9.648717
18	OE + FI-YES vs .OE + FI-NO	0.438578	9.648717
19	OE vs .OE + FI-BOTH	0.518605	9.648717
20	OE vs .CMAR	0.518605	9.648717
21	FI-BOTH vs .FI-YES	0.518605	9.648717

22	FI-BOTH vs .OE + FI-NO	0.518605	9.648717
23	FI-YES vs .CPAR	0.518605	9.648717
24	OE + FI-YES vs .FI-NO	0.518605	9.648717
25	OE + FI-BOTH vs .OE + FI-NO	0.605577	9.648717
26	FI-YES vs .OE + FI-YES	0.605577	9.648717
27	FI-NO vs .CPAR	0.605577	9.648717
28	OE + FI-NO vs .CMAR	0.605577	9.648717
29	OE + FI-BOTH vs .OE + FI-YES	0.796253	9.648717
30	OE + FI-YES vs .CMAR	0.796253	9.648717
31	OE vs .OE + FI-NO	0.897279	9.648717
32	FI-BOTH vs .OE + FI-BOTH	0.897279	9.648717
33	FI-BOTH vs .OE + FI-YES	0.897279	9.648717
34	FI-BOTH vs .CMAR	0.897279	9.648717
35	FI-YES vs .FI-NO	0.897279	9.648717
36	OE + FI-BOTH vs .CMAR	1	9.648717

Table S1: Adjusted p-values of algorithms having no feature selection.

i	hypothesis	unadjusted p	p_{Shaf}
1	OE + FI-NO + FI-CENTER + PCA-100 vs .OE FI-YES uni	0.000079	0.018252
2	OE FI-BOTH CENTER RFE vs .OE FI-YES uni	0.000736	0.154627
3	OE FI-YES uni vs .OE+RFE	0.000736	0.154627
4	OE + FI-NO + PCA-100 vs .OE + FI-NO + FI-CENTER + PCA-100	0.001317	0.276477
5	OE + FI-NO + FI-CENTER + PCA-100 vs .OE FI-BOTH CENTER uni	0.001447	0.303854
6	OE + FI-NO + FI-CENTER + PCA-100 vs .OE FI-BOTH uni	0.002513	0.527709
7	OE + FI-NO + FI-CENTER + PCA-100 vs .OE FI-YES CENTER uni	0.003004	0.630822
8	OE + FI-YES + FI-CENTER + PCA-100 vs .OE FI-YES uni	0.006482	1.361301

9	OE FI-YES CENTER RFE vs .OE FI-YES uni	0.006482	1.361301
10	OE vs .OE FI-YES uni	0.007037	1.477699
11	OE FI-YES uni vs .OE+UNI	0.007633	1.602957
12	OE + FI-BOTH + FI-CENTER + PCA-100 vs .OE FI-YES uni	0.008275	1.737651
13	OE + FI-NO + PCA-100 vs .OE FI-BOTH CENTER RFE	0.008275	1.737651
14	OE + FI-NO + PCA-100 vs .OE+RFE	0.008275	1.737651
15	OE FI-BOTH CENTER RFE vs .OE FI-BOTH CENTER uni	0.008964	1.882383
16	OE FI-BOTH CENTER uni vs .OE+RFE	0.008964	1.882383
17	OE FI-YES RFE vs .OE FI-YES uni	0.012261	2.574816
18	OE FI-NO CENTER RFE vs .OE FI-YES uni	0.012261	2.574816
19	OE + PCA-100 vs .OE FI-YES uni	0.013237	2.779852
20	OE FI-BOTH CENTER RFE vs .OE FI-BOTH uni	0.014282	2.999191
21	OE FI-BOTH uni vs .OE+RFE	0.014282	2.999191
22	OE FI-BOTH CENTER RFE vs .OE FI-YES CENTER uni	0.016591	3.484111
23	OE FI-YES CENTER uni vs .OE+RFE	0.016591	3.484111
24	OE + FI-BOTH + PCA-100 vs .OE FI-YES uni	0.019222	3.67136
25	OE + FI-YES + PCA-100 vs .OE FI-YES uni	0.02221	4.242133
26	OE FI-NO RFE vs .OE FI-YES uni	0.027448	5.242589
27	OE FI-BOTH RFE vs .OE FI-YES uni	0.043955	8.395351
28	OE FI-YES uni vs .OE FI-NO uni	0.043955	8.395351
29	OE FI-YES uni vs .OE FI-NO CENTER uni	0.043955	8.395351
30	OE + FI-YES + FI-CENTER + PCA-100 vs .OE + FI-NO + PCA-100	0.046889	8.955849
31	OE + FI-NO + PCA-100 vs .OE FI-YES CENTER RFE	0.046889	8.955849
32	OE vs .OE + FI-NO + PCA-100	0.049987	9.547503
33	OE + FI-YES + FI-CENTER + PCA-100 vs .OE FI-BOTH CENTER uni	0.049987	9.547503
34	OE FI-YES CENTER RFE vs .OE FI-BOTH CENTER uni	0.049987	9.547503
35	OE vs .OE FI-BOTH CENTER uni	0.053254	10.171584

36	OE + FI-NO + PCA-100 vs .OE+UNI	0.053254	10.171584
37	OE + FI-NO + FI-CENTER + PCA-100 vs .OE FI-BOTH RFE	0.053254	10.171584
38	OE + FI-NO + FI-CENTER + PCA-100 vs .OE FI-NO uni	0.053254	10.171584
39	OE + FI-NO + FI-CENTER + PCA-100 vs .OE FI-NO CENTER uni	0.053254	10.171584
40	OE + FI-BOTH + FI-CENTER + PCA-100 vs .OE + FI-NO + PCA-100	0.056698	10.829379
41	OE FI-BOTH CENTER uni vs .OE+UNI	0.056698	10.829379
42	OE + FI-BOTH + FI-CENTER + PCA-100 vs .OE FI-BOTH CENTER uni	0.060326	11.461873
43	OE + FI-YES + FI-CENTER + PCA-100 vs .OE FI-BOTH uni	0.072378	12.593687
44	OE FI-YES CENTER RFE vs .OE FI-BOTH uni	0.072378	12.593687
45	OE vs .OE FI-BOTH uni	0.076808	13.364648
46	OE + FI-NO + PCA-100 vs .OE FI-YES RFE	0.076808	13.364648
47	OE + FI-NO + PCA-100 vs .OE FI-NO CENTER RFE	0.076808	13.364648
48	OE + PCA-100 vs .OE + FI-NO + PCA-100	0.081458	14.173653
49	OE + FI-YES + FI-CENTER + PCA-100 vs .OE FI-YES CENTER uni	0.081458	14.173653
50	OE + FI-NO + FI-CENTER + PCA-100 vs .OE FI-NO RFE	0.081458	14.173653
51	OE FI-YES RFE vs .OE FI-BOTH CENTER uni	0.081458	14.173653
52	OE FI-YES CENTER RFE vs .OE FI-YES CENTER uni	0.081458	14.173653
53	OE FI-NO CENTER RFE vs .OE FI-BOTH CENTER uni	0.081458	14.173653
54	OE FI-BOTH uni vs .OE+UNI	0.081458	14.173653
55	OE vs .OE FI-YES CENTER uni	0.086333	15.021953
56	OE + PCA-100 vs .OE FI-BOTH CENTER uni	0.086333	15.021953
57	OE + FI-BOTH + FI-CENTER + PCA-100 vs .OE FI-BOTH uni	0.086333	15.021953
58	OE FI-YES CENTER uni vs .OE+UNI	0.091441	15.910796
59	OE + FI-BOTH + FI-CENTER + PCA-100 vs .OE FI-YES CENTER uni	0.09679	16.647851
60	OE + FI-YES + PCA-100 vs .OE + FI-NO + FI-CENTER + PCA-100	0.09679	16.647851
61	OE + FI-BOTH + PCA-100 vs .OE + FI-NO + PCA-100	0.108236	18.50833

62	OE + FI-BOTH + PCA-100 vs .OE + FI-NO + FI-CENTER + PCA-100	0.108236	18.50833
63	OE + FI-BOTH + PCA-100 vs .OE FI-BOTH CENTER uni	0.114348	18.50833
64	OE FI-YES RFE vs .OE FI-BOTH uni	0.114348	18.50833
65	OE FI-NO CENTER RFE vs .OE FI-BOTH uni	0.114348	18.50833
66	OE + PCA-100 vs .OE FI-BOTH uni	0.120728	19.195694
67	OE + FI-YES + PCA-100 vs .OE + FI-NO + PCA-100	0.120728	19.195694
68	OE + FI-YES + PCA-100 vs .OE FI-BOTH CENTER uni	0.127383	20.253898
69	OE FI-YES RFE vs .OE FI-YES CENTER uni	0.127383	20.253898
70	OE FI-NO CENTER RFE vs .OE FI-YES CENTER uni	0.127383	20.253898
71	OE + PCA-100 vs .OE FI-YES CENTER uni	0.13432	21.35694
72	OE + PCA-100 vs .OE + FI-NO + FI-CENTER + PCA-100	0.141546	22.50587
73	OE + FI-NO + PCA-100 vs .OE FI-NO RFE	0.141546	22.50587
74	OE + FI-NO + FI-CENTER + PCA-100 vs .OE FI-YES RFE	0.149067	23.254507
75	OE + FI-NO + FI-CENTER + PCA-100 vs .OE FI-NO CENTER RFE	0.149067	23.254507
76	OE FI-NO RFE vs .OE FI-BOTH CENTER uni	0.149067	23.254507
77	OE + FI-BOTH + PCA-100 vs .OE FI-BOTH uni	0.15689	24.317892
78	OE + FI-BOTH + PCA-100 vs .OE FI-YES CENTER uni	0.173462	26.713139
79	OE + FI-YES + PCA-100 vs .OE FI-BOTH uni	0.173462	26.713139
80	OE FI-BOTH RFE vs .OE FI-BOTH CENTER RFE	0.173462	26.713139
81	OE FI-BOTH RFE vs .OE+RFE	0.173462	26.713139
82	OE FI-BOTH CENTER RFE vs .OE FI-NO uni	0.173462	26.713139
83	OE FI-BOTH CENTER RFE vs .OE FI-NO CENTER uni	0.173462	26.713139
84	OE FI-NO uni vs .OE+RFE	0.173462	26.713139
85	OE FI-NO CENTER uni vs .OE+RFE	0.173462	26.713139
86	OE + FI-BOTH + FI-CENTER + PCA-100 vs .OE + FI-NO + FI-CENTER + PCA-100	0.191309	27.93108
87	OE + FI-YES + PCA-100 vs .OE FI-YES CENTER uni	0.191309	27.93108

88	OE + FI-NO + PCA-100 vs .OE FI-BOTH RFE	0.200723	28.50269
89	OE + FI-NO + PCA-100 vs .OE FI-NO uni	0.200723	28.50269
90	OE + FI-NO + PCA-100 vs .OE FI-NO CENTER uni	0.200723	28.50269
91	OE + FI-NO + FI-CENTER + PCA-100 vs .OE+UNI	0.200723	28.50269
92	OE FI-NO RFE vs .OE FI-BOTH uni	0.200723	28.50269
93	OE vs .OE + FI-NO + FI-CENTER + PCA-100	0.210471	29.255504
94	OE FI-BOTH RFE vs .OE FI-BOTH CENTER uni	0.210471	29.255504
95	OE FI-BOTH CENTER uni vs .OE FI-NO uni	0.210471	29.255504
96	OE FI-BOTH CENTER uni vs .OE FI-NO CENTER uni	0.210471	29.255504
97	OE + FI-YES + FI-CENTER + PCA-100 vs .OE + FI-NO + FI-CENTER + PCA-100	0.220557	29.775244
98	OE + FI-NO + FI-CENTER + PCA-100 vs .OE FI-YES CENTER RFE	0.220557	29.775244
99	OE FI-NO RFE vs .OE FI-YES CENTER uni	0.220557	29.775244
100	OE FI-BOTH CENTER RFE vs .OE FI-NO RFE	0.241759	31.428704
101	OE FI-NO RFE vs .OE+RFE	0.241759	31.428704
102	OE + FI-YES + PCA-100 vs .OE FI-BOTH CENTER RFE	0.276184	35.90396
103	OE + FI-YES + PCA-100 vs .OE+RFE	0.276184	35.90396
104	OE FI-BOTH RFE vs .OE FI-BOTH uni	0.276184	35.90396
105	OE FI-BOTH uni vs .OE FI-NO uni	0.276184	35.90396
106	OE FI-BOTH uni vs .OE FI-NO CENTER uni	0.276184	35.90396
107	OE + FI-BOTH + PCA-100 vs .OE FI-BOTH CENTER RFE	0.30091	37.312807
108	OE + FI-BOTH + PCA-100 vs .OE+RFE	0.30091	37.312807
109	OE FI-BOTH RFE vs .OE FI-YES CENTER uni	0.30091	37.312807
110	OE FI-YES CENTER uni vs .OE FI-NO uni	0.30091	37.312807
111	OE FI-YES CENTER uni vs .OE FI-NO CENTER uni	0.30091	37.312807
112	OE FI-YES uni vs .OE FI-YES CENTER uni	0.327067	39.248089
113	OE FI-BOTH uni vs .OE FI-YES uni	0.354658	42.204348

114	OE + PCA-100 vs .OE FI-BOTH CENTER RFE	0.368989	42.802764
115	OE + PCA-100 vs .OE+RFE	0.368989	42.802764
116	OE FI-BOTH CENTER RFE vs .OE FI-YES RFE	0.383675	44.50631
117	OE FI-BOTH CENTER RFE vs .OE FI-NO CENTER RFE	0.383675	44.50631
118	OE FI-YES RFE vs .OE+RFE	0.383675	44.50631
119	OE FI-NO CENTER RFE vs .OE+RFE	0.383675	44.50631
120	OE FI-BOTH CENTER uni vs .OE FI-YES uni	0.44591	49.941896
121	OE + FI-BOTH + FI-CENTER + PCA-100 vs .OE FI-BOTH CENTER RFE	0.462322	51.317762
122	OE + FI-BOTH + FI-CENTER + PCA-100 vs .OE+RFE	0.462322	51.317762
123	OE + FI-NO + PCA-100 vs .OE FI-YES uni	0.462322	51.317762
124	OE + FI-YES + FI-CENTER + PCA-100 vs .OE FI-BOTH RFE	0.479066	51.739156
125	OE + FI-YES + FI-CENTER + PCA-100 vs .OE FI-NO uni	0.479066	51.739156
126	OE + FI-YES + FI-CENTER + PCA-100 vs .OE FI-NO CENTER uni	0.479066	51.739156
127	OE FI-BOTH RFE vs .OE FI-YES CENTER RFE	0.479066	51.739156
128	OE FI-BOTH CENTER RFE vs .OE+UNI	0.479066	51.739156
129	OE FI-YES CENTER RFE vs .OE FI-NO uni	0.479066	51.739156
130	OE FI-YES CENTER RFE vs .OE FI-NO CENTER uni	0.479066	51.739156
131	OE+RFE vs .OE+UNI	0.479066	51.739156
132	OE vs .OE FI-BOTH RFE	0.496136	51.739156
133	OE vs .OE FI-BOTH CENTER RFE	0.496136	51.739156
134	OE vs .OE FI-NO uni	0.496136	51.739156
135	OE vs .OE FI-NO CENTER uni	0.496136	51.739156
136	OE vs .OE+RFE	0.496136	51.739156
137	OE + FI-YES + FI-CENTER + PCA-100 vs .OE FI-BOTH CENTER RFE	0.513525	51.739156
138	OE + FI-YES + FI-CENTER + PCA-100 vs .OE+RFE	0.513525	51.739156
139	OE FI-BOTH RFE vs .OE+UNI	0.513525	51.739156

140	OE FI-BOTH CENTER RFE vs .OE FI-YES CENTER RFE	0.513525	51.739156
141	OE FI-YES CENTER RFE vs .OE+RFE	0.513525	51.739156
142	OE FI-NO uni vs .OE+UNI	0.513525	51.739156
143	OE FI-NO CENTER uni vs .OE+UNI	0.513525	51.739156
144	OE + FI-BOTH + FI-CENTER + PCA-100 vs .OE FI-BOTH RFE	0.531226	51.739156
145	OE + FI-BOTH + FI-CENTER + PCA-100 vs .OE FI-NO uni	0.531226	51.739156
146	OE + FI-BOTH + FI-CENTER + PCA-100 vs .OE FI-NO CENTER uni	0.531226	51.739156
147	OE + FI-NO + FI-CENTER + PCA-100 vs .OE FI-BOTH CENTER RFE	0.567533	51.739156
148	OE + FI-NO + FI-CENTER + PCA-100 vs .OE+RFE	0.567533	51.739156
149	OE + FI-YES + FI-CENTER + PCA-100 vs .OE FI-NO RFE	0.604987	51.739156
150	OE FI-YES CENTER RFE vs .OE FI-NO RFE	0.604987	51.739156
151	OE vs .OE FI-NO RFE	0.62412	51.739156
152	OE FI-BOTH RFE vs .OE FI-YES RFE	0.62412	51.739156
153	OE FI-BOTH RFE vs .OE FI-NO CENTER RFE	0.62412	51.739156
154	OE FI-YES RFE vs .OE FI-NO uni	0.62412	51.739156
155	OE FI-YES RFE vs .OE FI-NO CENTER uni	0.62412	51.739156
156	OE FI-NO CENTER RFE vs .OE FI-NO uni	0.62412	51.739156
157	OE FI-NO CENTER RFE vs .OE FI-NO CENTER uni	0.62412	51.739156
158	OE + PCA-100 vs .OE FI-BOTH RFE	0.643511	51.739156
159	OE + PCA-100 vs .OE FI-NO uni	0.643511	51.739156
160	OE + PCA-100 vs .OE FI-NO CENTER uni	0.643511	51.739156
161	OE FI-NO RFE vs .OE+UNI	0.643511	51.739156
162	OE + FI-BOTH + FI-CENTER + PCA-100 vs .OE FI-NO RFE	0.663147	51.739156
163	OE + FI-YES + PCA-100 vs .OE + FI-YES + FI-CENTER + PCA-100	0.663147	51.739156
164	OE + FI-YES + PCA-100 vs .OE FI-YES CENTER RFE	0.663147	51.739156
165	OE vs .OE + FI-YES + PCA-100	0.683017	51.739156
166	OE + FI-BOTH + PCA-100 vs .OE + FI-YES + FI-CENTER + PCA-100	0.70311	51.739156

167	OE + FI-BOTH + PCA-100 vs .OE FI-YES CENTER RFE	0.70311	51.739156
168	OE + FI-YES + PCA-100 vs .OE+UNI	0.70311	51.739156
169	OE vs .OE + FI-BOTH + PCA-100	0.723412	51.739156
170	OE + FI-BOTH + FI-CENTER + PCA-100 vs .OE + FI-YES + PCA-100	0.723412	51.739156
171	OE + FI-BOTH + PCA-100 vs .OE FI-BOTH RFE	0.74391	51.739156
172	OE + FI-BOTH + PCA-100 vs .OE FI-NO uni	0.74391	51.739156
173	OE + FI-BOTH + PCA-100 vs .OE FI-NO CENTER uni	0.74391	51.739156
174	OE + FI-BOTH + PCA-100 vs .OE+UNI	0.74391	51.739156
175	OE + FI-BOTH + PCA-100 vs .OE + FI-BOTH + FI-CENTER + PCA-100	0.764592	51.739156
176	OE FI-YES RFE vs .OE FI-NO RFE	0.764592	51.739156
177	OE FI-NO RFE vs .OE FI-NO CENTER RFE	0.764592	51.739156
178	OE + PCA-100 vs .OE FI-NO RFE	0.785443	51.739156
179	OE + FI-YES + PCA-100 vs .OE FI-BOTH RFE	0.785443	51.739156
180	OE + FI-YES + PCA-100 vs .OE FI-NO uni	0.785443	51.739156
181	OE + FI-YES + PCA-100 vs .OE FI-NO CENTER uni	0.785443	51.739156
182	OE + PCA-100 vs .OE + FI-YES + FI-CENTER + PCA-100	0.806449	51.739156
183	OE + PCA-100 vs .OE FI-YES CENTER RFE	0.806449	51.739156
184	OE + FI-NO + PCA-100 vs .OE FI-YES CENTER uni	0.806449	51.739156
185	OE vs .OE + PCA-100	0.827596	51.739156
186	OE + FI-YES + PCA-100 vs .OE FI-YES RFE	0.827596	51.739156
187	OE + FI-YES + PCA-100 vs .OE FI-NO CENTER RFE	0.827596	51.739156
188	OE + FI-YES + FI-CENTER + PCA-100 vs .OE FI-YES RFE	0.827596	51.739156
189	OE + FI-YES + FI-CENTER + PCA-100 vs .OE FI-NO CENTER RFE	0.827596	51.739156
190	OE FI-YES RFE vs .OE FI-YES CENTER RFE	0.827596	51.739156
191	OE FI-YES CENTER RFE vs .OE FI-NO CENTER RFE	0.827596	51.739156
192	OE FI-BOTH CENTER uni vs .OE FI-YES CENTER uni	0.827596	51.739156

193	OE vs .OE FI-YES RFE	0.848868	51.739156
194	OE vs .OE FI-NO CENTER RFE	0.848868	51.739156
195	OE + PCA-100 vs .OE + FI-YES + PCA-100	0.848868	51.739156
196	OE + PCA-100 vs .OE+UNI	0.848868	51.739156
197	OE + FI-NO + PCA-100 vs .OE FI-BOTH uni	0.848868	51.739156
198	OE FI-BOTH RFE vs .OE FI-NO RFE	0.848868	51.739156
199	OE FI-NO RFE vs .OE FI-NO uni	0.848868	51.739156
200	OE FI-NO RFE vs .OE FI-NO CENTER uni	0.848868	51.739156
201	OE + PCA-100 vs .OE + FI-BOTH + FI-CENTER + PCA-100	0.870251	51.739156
202	OE + FI-BOTH + PCA-100 vs .OE FI-YES RFE	0.870251	51.739156
203	OE + FI-BOTH + PCA-100 vs .OE FI-NO CENTER RFE	0.870251	51.739156
204	OE FI-YES RFE vs .OE+UNI	0.870251	51.739156
205	OE FI-NO CENTER RFE vs .OE+UNI	0.870251	51.739156
206	OE FI-BOTH uni vs .OE FI-BOTH CENTER uni	0.870251	51.739156
207	OE + PCA-100 vs .OE + FI-BOTH + PCA-100	0.891729	51.739156
208	OE + FI-BOTH + PCA-100 vs .OE FI-NO RFE	0.891729	51.739156
209	OE + FI-BOTH + FI-CENTER + PCA-100 vs .OE FI-YES RFE	0.891729	51.739156
210	OE + FI-BOTH + FI-CENTER + PCA-100 vs .OE FI-NO CENTER RFE	0.891729	51.739156
211	OE + FI-BOTH + FI-CENTER + PCA-100 vs .OE + FI-YES + FI-CENTER + PCA-100	0.934909	51.739156
212	OE + FI-BOTH + FI-CENTER + PCA-100 vs .OE FI-YES CENTER RFE	0.934909	51.739156
213	OE + FI-YES + PCA-100 vs .OE FI-NO RFE	0.934909	51.739156
214	OE vs .OE + FI-BOTH + FI-CENTER + PCA-100	0.956579	51.739156
215	OE + FI-BOTH + PCA-100 vs .OE + FI-YES + PCA-100	0.956579	51.739156
216	OE + FI-YES + FI-CENTER + PCA-100 vs .OE+UNI	0.956579	51.739156
217	OE FI-YES CENTER RFE vs .OE+UNI	0.956579	51.739156
218	OE FI-BOTH uni vs .OE FI-YES CENTER uni	0.956579	51.739156

219	OE vs .OE + FI-YES + FI-CENTER + PCA-100	0.978282	51.739156
220	OE vs .OE FI-YES CENTER RFE	0.978282	51.739156
221	OE vs .OE+UNI	0.978282	51.739156
222	OE + PCA-100 vs .OE FI-YES RFE	0.978282	51.739156
223	OE + PCA-100 vs .OE FI-NO CENTER RFE	0.978282	51.739156
224	OE + FI-BOTH + FI-CENTER + PCA-100 vs .OE+UNI	0.978282	51.739156
225	OE + FI-NO + PCA-100 vs .OE FI-BOTH CENTER uni	0.978282	51.739156
226	OE + FI-YES + FI-CENTER + PCA-100 vs .OE FI-YES CENTER RFE	1	51.739156
227	OE FI-BOTH RFE vs .OE FI-NO uni	1	51.739156
228	OE FI-BOTH RFE vs .OE FI-NO CENTER uni	1	51.739156
229	OE FI-BOTH CENTER RFE vs .OE+RFE	1	51.739156
230	OE FI-YES RFE vs .OE FI-NO CENTER RFE	1	51.739156
231	OE FI-NO uni vs .OE FI-NO CENTER uni	1	51.739156

Table S2: Adjusted p-values of algorithms having feature selection.
